# DeepLPI: a multimodal deep learning method for predicting the interactions between lncRNAs and protein isoforms

**DOI:** 10.1186/s12859-020-03914-7

**Published:** 2021-01-18

**Authors:** Dipan Shaw, Hao Chen, Minzhu Xie, Tao Jiang

**Affiliations:** 1 Department of Computer Science and Engineering, University of California, Riverside, CA 92521 USA; 2 College of Information Science and Engineering, Hunan Normal University, Changsha, China; 3 Bioinformatics Division, BNRIST/Department of Computer Science and Technology, Tsinghua University, Beijing, China

## Abstract

**Background:**

Long non-coding RNAs (lncRNAs) regulate diverse biological processes via interactions with proteins. Since the experimental methods to identify these interactions are expensive and time-consuming, many computational methods have been proposed. Although these computational methods have achieved promising prediction performance, they neglect the fact that a gene may encode multiple protein isoforms and different isoforms of the same gene may interact differently with the same lncRNA.

**Results:**

In this study, we propose a novel method, DeepLPI, for predicting the interactions between lncRNAs and protein isoforms. Our method uses sequence and structure data to extract intrinsic features and expression data to extract topological features. To combine these different data, we adopt a hybrid framework by integrating a multimodal deep learning neural network and a conditional random field. To overcome the lack of known interactions between lncRNAs and protein isoforms, we apply a multiple instance learning (MIL) approach. In our experiment concerning the human lncRNA-protein interactions in the NPInter v3.0 database, DeepLPI improved the prediction performance by 4.7% in term of AUC and 5.9% in term of AUPRC over the state-of-the-art methods. Our further correlation analyses between interactive lncRNAs and protein isoforms also illustrated that their co-expression information helped predict the interactions. Finally, we give some examples where DeepLPI was able to outperform the other methods in predicting mouse lncRNA-protein interactions and novel human lncRNA-protein interactions.

**Conclusion:**

Our results demonstrated that the use of isoforms and MIL contributed significantly to the improvement of performance in predicting lncRNA and protein interactions. We believe that such an approach would find more applications in predicting other functional roles of RNAs and proteins.

## Background

Long non-coding RNAs (lncRNAs) are RNA transcripts of more than 200 nucleotides that are not translated to proteins. Previous research [1, 2] has demonstrated that lncRNAs participate energetically in almost the whole process of cells. However, the functions of most lncRNAs are unknown. To understand the function of an lncRNA, it is necessary to identify what other biological molecules it is able to interact with, especially proteins [3, 4]. By interacting with proteins, lncRNAs could regulate the expression of genes, influence nuclear architecture and modulate the activity of proteins [5]. Therefore, identifying lncRNA-protein interactions is an important approach to understand the potential functions of lncRNAs.

Current methods to identify lncRNA-protein interactions are based on biological experiments and computational models. With the rapid development of molecular biology techniques, large-scale experimental approaches such as PAR-CLIP [6], RNAcompete [7], HITS-CLIP [8], and RIP-Chip [9] have been developed to detect RNA-protein binding and have been used to find lncRNA-protein interactions. However, these experimental approaches are expensive and time-consuming [3]. Based on the known lncRNA-protein interactions, many computational methods have been introduced for mining novel lncRNA-protein interactions. According to Zhang et al. [3], the computational methods could be grouped into two broad categories, machine learning-based methods and network-based methods. The machine learning-based methods build binary classifiers to predict lncRNA-protein pairs as interactive or non-interactive. These methods trained their classifiers using sequence, structure and physicochemical features of lncRNAs and proteins. For example, RPISeq [10] utilized the sequence information of RNAs and proteins to train a random forest classifier and a support vector machine classifier. Bellucci et al. trained catRAPID [11] using the physicochemical properties and secondary structure propensities of 592 protein-RNA pairs to predict novel RNA-protein interactions. Wang et al. [12] built a protein-RNA interaction prediction model using the naive Bayes classifier based only on sequence information. LncPro [13] used Fisher’s linear discriminant approach to compute a matrix based on lncRNA and protein sequence information, and used the matrix to score the interactions between an lncRNA-protein pair. Based on the sequence and secondary structural information of RNAs and proteins, RPI-Pred [14] trained a support vector machine. RpiCOOL [15] trained a random forest classifier using sequence motifs and repeat patterns. LPI-BLS [16] used sequence information of known lncRNA-protein pairs to learn multiple BLS (broad learning system) classifiers and integrated the classifiers with a logistical regression model. Recently, IPMiner [17], RPI-SAN [18], RPITER [19] and lncADeep [20] employed deep learning techniques to build lncRNA-protein interaction prediction models based on sequence and/or structural information.

Note that, there are several recently developed methods for predicting general ncRNA-protein interactions based on machine learning [21–24], but they do not consider lncRNAs specifically and are hence less relevant to our work.

The network-based methods integrate heterozygous information associated with lncRNAs and proteins into a network  [3] and utilize the topological relationship of lncRNAs and proteins to predict lncRNA-protein interactions. Li et al. proposed LPIHN [25] that integrated an lncRNA-lncRNA similarity network, an lncRNA-protein interaction network and a protein–protein interaction (PPI) network into a heterogeneous network, and used a random walk with restart technique on the heterogeneous network to infer lncRNA-protein interactions. Ge et al. developed a different network approach LPBNI [26] using an lncRNA-protein bipartite network inference method. Based on a heterogenous network similar to LPIHN, Xiao et al. proposed PLPIHS [27] using HeteSim scores [28] to infer lncRNA-protein interactions, and Hu et al. introduced an eigenvalue transformation-based semi-supervised link prediction method LPI-ETSLP [29]. Zhang et al. designed a linear neighborhood propagation method LPLNP [30]. Zhao et al. utilized both random walk and neighborhood regularized logistic matrix factorization and proposed IRWNRLPI [31]. Deng et al. proposed PLIPCOM [32], which combined diffusion and HeteSim features of heterogeneous lncRNA-protein networks and applied a gradient tree boosting algorithm to predict interactions. More recently, [33] combined multiple similarities and multiple features related to lncRNAs and proteins into a feature projection ensemble learning frame. Zhao et al. proposed a semi-supervised learning method LPI-BNPRA [34]. Shen et al. proposed LPI-KTASLP [35], which used multivariate information about lncRNAs and proteins to conduct a semi-supervised link prediction. Xie et al. [36] constructed a network integrating the information about lncRNA expressions, protein–protein interactions and known lncRNA-protein interactions, and adopted a bipartite network recommendation method to predict lncRNA-protein interactions.

Though a lot of computational methods for predicting lncRNA-protein interactions have been introduced, many challenges still remain. First, in the above studies, the machine-learning based methods only focused on the intrinsic features of lncRNAs and proteins and the network based methods mostly focused on the topological features of associated biological networks of lncRNAs and proteins [3]. An integration of all these features might lead to a better prediction. Second, all methods proposed above neglected the fact that a gene may encode multiple protein isoforms and different isoforms of the same gene may interact differently with the same lncRNA, which could inevitably impact their prediction performance. In this paper, we attempt to address these issues and propose a novel method, named DeepLPI (multimodal **Deep** learning method for predicting LncRNA-Protein Isoform interactions). DeepLPI uses sequence, structure and expression data of lncRNAs and protein isoforms. Instead of using the canonical proteins of each gene, DeepLPI considers all protein isoforms, which could help to detect lncRNA-protein interactions more accurately. DeepLPI extracts intrinsic features such as functional motifs from the sequence and structure data, and obtains network topological features from the expression data. Note that, DeepLPI uses mRNA expression data to extract network topological features instead of PPI data as done in the existing methods because most of the available PPI data do not provide the details about isoforms. Moreover, it is possible to build an isoform-isoform interaction network based on mRNA expression data [37].

DeepLPI consists of two parts. In the first part, we train a deep neural network (DNN) that uses the multimodal deep learning (MDL) [38] technique to extract features from the sequence and structure data of lncRNAs and protein isoforms. The MDL fuses these extracted features and measures the initial interaction scores between lncRNAs and protein isoforms. In the second part, a conditional random field (CRF) is designed to exploit the co-expression relationship among lncRNAs and the co-expression relationship among protein isoforms. The CRF assigns final interaction scores between lncRNAs and protein isoforms based on initial interaction scores while trying to keep highly co-expressed lncRNAs and highly co-expressed protein isoforms attaining similar interaction patterns. To overcome the lack of interaction training labels for lncRNAs and protein isoforms, we propose an iterative semi-supervised training algorithm based on the multiple instance learning (MIL) framework similar to [39–41]. In MIL, for each positive lncRNA and protein interaction pair (*r*, *p*), we initially assign positive interaction labels to all pairs (*r*, *i*) for each isoform *i* of *p* and negative interaction labels to all other pairs of lncRNAs and protein isoforms. In each iteration, the DNN and CRF update the initial interaction scores using co-expressed lncRNAs and co-expressed isoforms until convergence is reached. In this setting, the isoforms of the a protein/gene can interact differently with the same lncRNA. This flexibility and the integration of both intrinsic and network topological features may potentially lead to a better prediction.

To evaluate the performance of DeepLPI, we first measure its prediction performance using protein (i.e., gene) level interactions with lncRNAs provided in the NPInter v3.0 database. We make sure at least a half of our negative interaction examples contain lncRNAs and proteins that are present in the positive interactions (but do not interact with each other). The rest of the negative interactions contain lncRNAs or proteins that are not present in the positive interactions. This helps overcome the overfitting issue. DeepLPI achieved an average AUC (area under receiver operating characteristic curve) of 0.866 and AUPRC (area under the precision-recall curve) of 0.703 on the human interaction dataset. We also compare our method with both machine-learning based methods and network based methods for predicting lncRNA-protein interactions surveyed above on the same dataset. Based on availability and ease of use, 11 methods were chosen for the comparison. The experimental results demonstrate that our method significantly outperformed the others. We further evaluate the effect of various components of our model (i.e., the so-called ablation study), which essentially indicates the effectiveness of each source of data (isoforms, structures, sequences, and expressions) incorporated and how these data are effectively captured by their corresponding components of the model. We also analyze the divergence of isoform interactions, i.e., how isoforms from the same protein may interact differently with lncRNAs. Finally, we validate our method via a series of tests including the correlation similarity test, prediction of mouse lncRNA-protein interactions using the model trained on human data (since lncRNAs are conserved), and case studies of recently discovered lncRNA-protein interactions in the literature.

## Results and Discussion

In this section, we first compare the performance of DeepLPI with some state-of-the-art methods and analyze the effectiveness of our method in terms of each type of data we used and each component of the model. Next, we validate the prediction results of DeepLPI using correlation analyses, a mouse dataset as well as some newly discovered human lncRNA-protein interactions in the literature.

### Prediction of lncRNA-protein interactions

We first compare the performance of DeepLPI with both of machine-learning based methods and network based methods. Then, we evaluate the effectiveness of each component of our model (i.e., the ablation study). We also study the divergence of lncRNAs interacting with different isoforms of the same protein/gene, and compare the structural components of lncRNAs and protein isoforms in both interactive and non-interactive pairs. Finally, we test DeepLPI on some smaller and older lncRNA-protein interaction datasets and observe how its performance could be impacted by the size of training data.

#### Prediction performance comparison between DeepLPI and the existing methods

Since there is no benchmark data for lncRNA-protein isoform interactions, we could only evaluate the performance of DeepLPI based on the benchmark data of (human) lncRNA-protein interactions downloaded from the NPInter v3.0 [42] database. We compare DeepLPI with some state-of-the-art methods including machine-learning based and network based methods.

The popular machine-learning based methods are catRAPID [11], RPISeq [10], lncPro [13], RPI-Pred [14], rpiCOOL [15], IPMiner [17], RPI-SAN [18], lncADeep [20], RPITER [19] and LPI-BLS [16]. Among these methods, some (lncPro and rpiCOOL) provide stand-alone programs, some (catRAPID, RPISeq and RPI-Pred) provide web-based services, some (IPMiner, lncADeep, RPITER and LPI-BLS) are re-trainable with available source codes, while the others are unavailable. Predicting lncRNA-protein interactions on a large scale using web-based services of catRAPID and RPI-Pred is time-consuming and often fails in the case of long input sequences. The publicly available network based methods are LPIHN [25], LPBNI [26], PLPIHS [43], LPLNP [30], PLIPCOM [32], and SFPEL-LPI [33]. Therefore, we compare our method with seven machine-learning based methods lncPro, rpiCOOL, IPMiner, lncADeep, RPITER, LPI-BLS and RPISeq, and six network based methods LPIHN, LPBNI, PLPIHS, LPLNP, PLIPCOM, and SFPEL-LPI. Default parameters of these methods are used as recommended by their authors.

Table 1 shows the average test results in 10 runs of five-fold cross validations on the NPInter v3.0 human dataset. The AUC values of RPISeq (RF), RPISeq (SVM), lncPro, rpiCOOL, IPMiner, lncADeep, RPITER, LPI-BLS and DeepLPI are 0.708, 0.701, 0.723, 0.721, 0.714, 0.825, 0.827, 0.782 and 0.866, respectively, and their AUPRC values are 0.486, 0.473, 0.588, 0.503, 0.569, 0.646, 0.664, 0.575 and 0.685, respectively. DeepLPI outperformed these machine-learning based methods by 22.3%, 23.5%, 19.7%, 20.1%, 21.2%, 4.9%, 4.7%, and 10.7% in terms of AUC and by 44.6%, 48.6%, 19.6%, 39.8%, 23.6%, 8.8%, 5.9%, and 22.2% in terms of AUPRC, respectively. The AUC values of LPIHN, LPBNI, PLPIHS, LPLNP, PLIPCOM and SFPEL-LPI are 0.776, 0.786, 0.672, 0.801, 0.821 and 0.823, respectively, and the AUPRC values are 0.421, 0.559, 0.483, 0.566, 0.609 and 0.599, respectively. DeepLPI also outperformed these network based methods by 11.6%, 10.2%, 28.9%, 8.1%, 5.5% and 5.2% in terms of AUC and 67.0%, 25.8%, 45.5%, 24.2%, 15.4% and 17.4% in terms of AUPRC scores, respectively. Since these results show that DeepLPI, lncADeep and RPITER performed better than the others, we will only compare these three methods in the following experiments.

**Table 1:** Comparison of prediction performance on lncRNA-protein interactions on the NPInter v3.0 human dataset

Broad category	Methods	AUC	AUPRC
Machine-learning based methods	RPISeq (RF)	0.708	0.486
	RPISeq (SVM)	0.701	0.473
	lncPro	0.723	0.588
	rpiCOOL	0.721	0.503
	IPMiner	0.714	0.569
	lncADeep	0.825	0.646
	RPITER	0.827	0.664
	LPI-BLS	0.782	0.575
	DeepLPI	0.866	0.703
Network based methods	LPIHN	0.776	0.421
	LPBNI	0.786	0.559
	PLPIHS	0.672	0.483
	LPLNP	0.801	0.566
	PLIPCOM	0.821	0.609
	SFPEL-LPI	0.823	0.599

To test if our sampling method for generating negative interactions is helpful in reducing overfitting, we repeat the above experiment with all negative interactions sampled randomly and compare DeepLPI with two of the best-performing existing methods, lncADeep and RPITER. The AUC values of DeepLPI, lncADeep and RPITER are 0.923, 0.905 and 0.894, respectively, and their AUPRC values are 0.776, 0.753 and 0.747, respectively. While all AUC and AUPRC values of the three methods have increased significantly, DeepLPI consistently performs better than the other two.

We also evaluate the performance of the methods using the leave-one-out cross-validation (LOOCV) experiment, although it is computationally more expensive. In this experiment, the AUC values of DeepLPI, lncADeep and RPITER are 0.855, 0.801 and 0.811, respectively, and their AUPRC values are 0.694, 0.638 and 0.649, respectively. Compared to those in the five-fold cross-validation experiment, the AUC and AUPRC values of all methods decreased a little, which might be due to variance in the data as discussed in [44].

In order to test if homologous protein sequences might have an impact on the performance of DeepLPI and potentially cause data leak and/or model overfitting, we search for homologous proteins in our benchmark dataset based on EggNog [45]. It turns out that only 5% of the proteins are homologous (to other sequences). We repeat the above five-fold cross-validation experiment for DeepLPI by keeping all interactions involving homologous proteins in the same fold. The AUC and AUPRC values decrease only slightly from 0.866 to 0.861 and from 0.703 to 0.699. This suggests that data leak or model overfitting were unlikely or very limited in our experiment.

#### Analyzing the effects of model components

In order to assess the contribution of the biological features considered in our model as well as its major computational components, we conduct an ablation study by removing various features/components from the model and evaluate how such a change would affect the performance of the model. More specifically, we test how the model is affected when the MIL learning with protein isoforms is replaced by conventional learning with proteins, when the CRF component along with the expression data are removed, and when the sequence or structure data are removed.

**Fig. 1:**
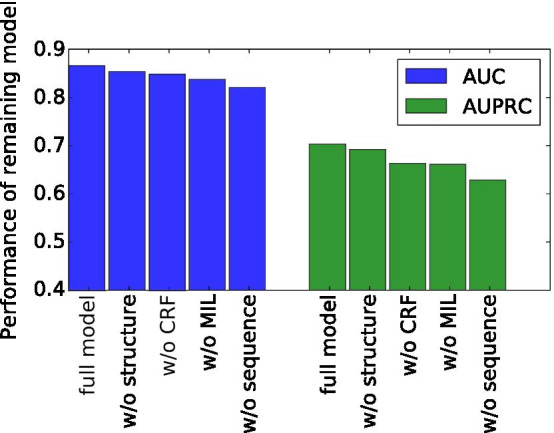
The effect on performance of removing various components from the model. The average AUC and AUPRC values of DeepLPI, DeepLPI without structure data, DeepLPI without CRF, DeepLPI without using MIL/isoforms, and DeepLPI without sequence data are shown in the figure

Figure 1 shows that the average AUC of DeepLPI dropped 1.4%, 2.0%, 3.3%, and 5.5% without the structure data, without the CRF component for incorporating expression data, without the MIL learning framework for incorporating isoforms, and without the sequence data, respectively. Without these components or data, the performance in term of AUPRC shows a similar declining trend with the percentage decreases being 1.6%, 6.0%, 6.3% and 11.6%, respectively. In particular, when we consider proteins instead of protein isoforms in the model, its AUC dropped from 0.866 to 0.842, which demonstrates the significance of using isoform data. The results also suggest that the CRF component was effective in improving the prediction performance via the integration of expression data. Among all types of input data, the sequences are clearly the most important for the model. Although the usage of structure data did not boost the performance of the model significantly, it allows us to observe interesting enrichment of structural components in interactive lncRNAs, as discussed in the next subsection.

#### Structural components at important positions in interactive and non-interactive pairs

**Fig. 2:**
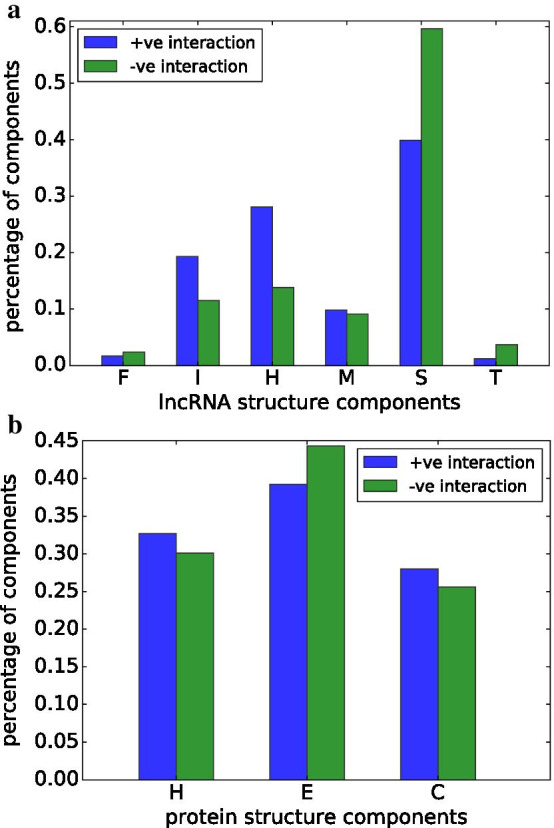
**a** Distribution of lncRNA structural components. **b** Distribution of protein structural components

It would be interesting to study how the structural components of lncRNAs and protein isoforms are distributed in interactive pairs, especially around their interacting sites, and what structural components may contribute more to the interactions than the others. For each lncRNA-protein isoform pair, we use saliency maps [46] to compute importance weights at each position in both sequences. These weights indicate how a position might impact the prediction outcome by our model (i.e., interactive or non-interactive). The lncRNA and protein structural components at heavily weighted (i.e., important) positions of interactive and non-interactive pairs are profiled and shown in Fig. 2a. For each structural component, the average occurrence frequency across all instances is calculated. We can see that at important lncRNA positions, hairpin loops (H) occur much more often in interactive pairs than in non-interactive pairs. The same appears to be true for inner loops (I). On the other hand, stems (S) occur much often at the important lncRNA positions of interactive pairs than at the important lncRNA positions of non-interactive pairs. These suggest that open/unpaired lncRNA positions perhaps play more important roles in their interactions with proteins, and is consistent with several studies in the literature [4, 47].

Similar to lncRNA structural components, we also profile protein isoform structural components in Fig. 2b. However, we are unable to observe a significant difference between the distributions of the structural components at important protein isoform positions of interactive and non-interactive pairs. We suspect that a more detailed protein structure representation might help reveal some difference, but was unable to pursue it given the time complexity involved in obtaining such representations with high quality.

#### Divergence of lncRNAs interacting with the isoforms of the same protein

Our ultimate goal is to find lncRNA interactions at the isoform level. Hence, it would be useful to analyze how lncRNAs interact divergently with the isoforms of the same protein. We first estimate the similarity of predicted lncRNA interactions for each pair of isoforms in terms of the semantic similarity score using GOssTo [48]. As in [39, 49], the semantic dissimilarity score between two isoforms is then defined as one minus their similarity score. We consider only proteins with multiple isoforms (MIPs) and collected all interactions between lncRNAs and the isoforms of the MIPs as predicted by DeepLPI trained on the the NPInter v3.0 dataset. For each MIP, the interaction divergence of its isoforms was calculated by averaging the semantic dissimilarity scores of all pairs of its isoforms. Among these MIPs, 71.6% (1548 out of 2163) were estimated to have divergent isoform interactions (i.e., with semantic dissimilarity scores greater than 0). The dissimilarity score distributions for MIPs that have divergent isoform interactions are shown in Additional file 1: Fig. S1 where the mean score value is 0.302.

#### The impact of training data size

We have collected several (older and smaller) ncRNA-protein interaction datasets including RPI369, RPI1807, RPI2241, and NPInter v2.0. We would like to test how the DeepLPI, lncADeep and RPITER methods perform when these different datasets are used for training and the comparatively newer dataset NPInter v3.0 is used for testing. Since the datasets overlap with each other quite a bit, we make sure that the test interactions do not contain any of the training interactions to prevent a possible data leak. The prediction results are shown in Table 2. The results suggests that the sample size of the training data has a significant effect on the prediction performance of DeepLPI, lncADeep and RPITER. When more training samples are available, these models achieve a better prediction performance, as expected. However, the rate of improvement with respect to the number of interactions is much higher for DeepLPI than for the other two methods.

**Table 2:**
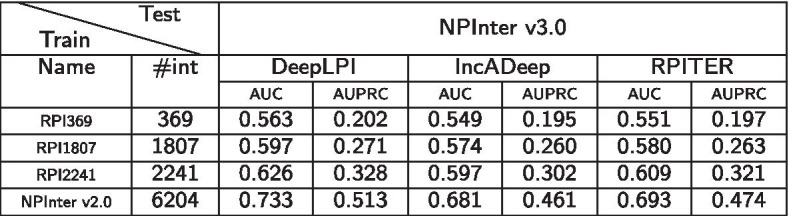
Performance of DeepLPI, lncADeep and RPITER when datasets from RPI369, RPI1807, RPI2241, and NPInter v2.0 are used for training and the NPInter v3.0 dataset is used for testing

Here, #int represents the number of positive lncRNA-protein interactions contained in a training dataset. As the training data increases, the performance DeepLPI, lncADeep and RPITER improves as expected, but the rate of improvement for DeepLPI is higher than the other methods

### Validation of predicted lncRNA-protein isoform interactions

To validate the prediction lncRNA-protein isoform interaction results of DeepLPI, we analyze the correlations between isoform sequence similarity, lncRNA sequence similarity, as well as their structure similarity and expression similarity. Moreover, we evaluate the prediction performance of DeepLPI (trained on the NPInter v3.0 human interaction data) using a mouse lncRNA-protein interaction dataset and some new human lncRNA-protein interactions from the recent literature that were not included in the NPInter v3.0 database as the test data.

#### Correlation analyses

Our basic assumption is that similar lncRNAs tend to interact with similar protein isoforms. To check if our predicted interactions accord to the assumption, we conducted a series analyses of correlation between the similarity of lncRNAs and the similarity of their interactive protein isoforms.

**Fig. 3:**
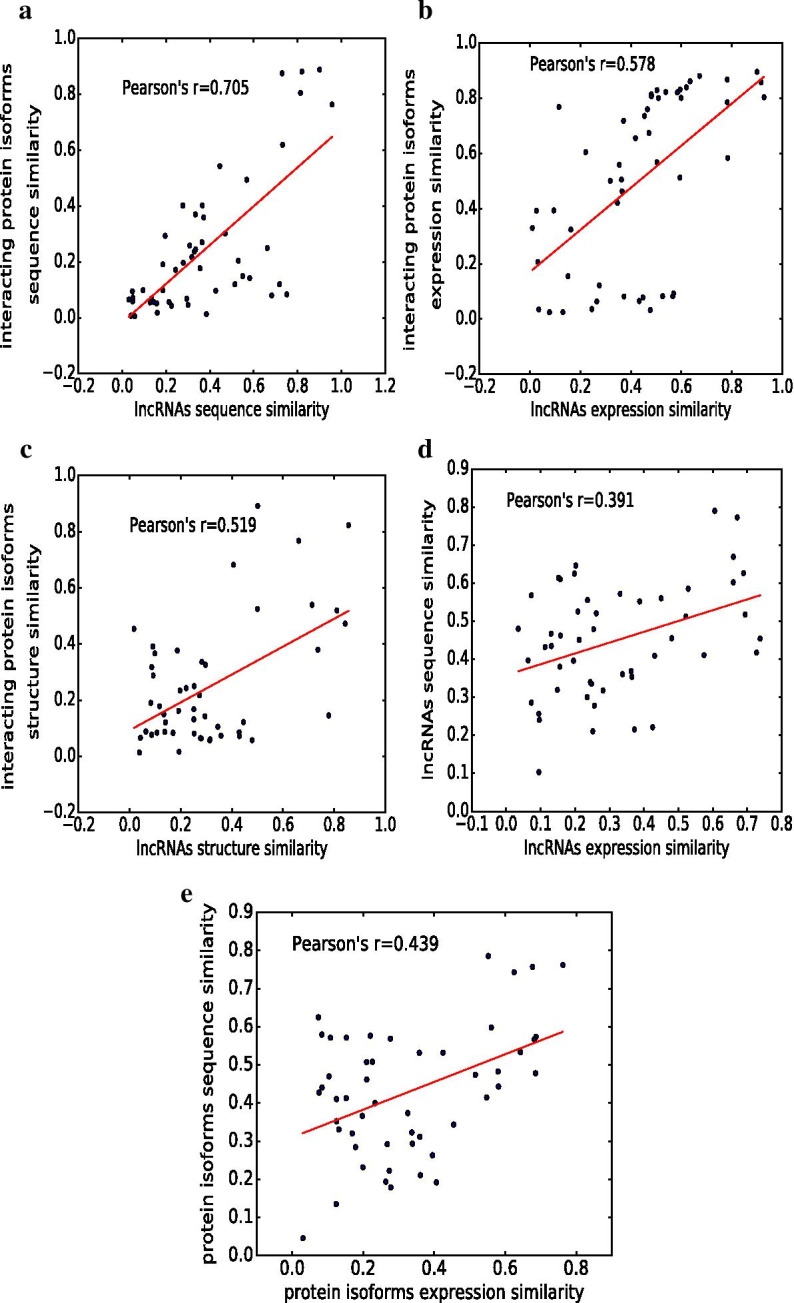
Correlation analysis. **a** Correlation between lncRNA sequence and protein isoform sequence similarities. **b** Correlation between lncRNA expression and protein isoform expression similarities. **c** Correlation between lncRNAs structure and protein isoform structure similarities. **d** Correlation between lncRNAs sequence and lncRNAs expression similarities. **e** Correlation between protein isoforms sequence and protein isoforms expression similarities

From the lncRNA-protein isoform interactions predicted by DeepLPI, we grouped 1,534 involved lncRNAs into 50 clusters according to a hierarchical clustering based on a generalized Levenshtein (edit) distance. For each group, we calculated a (sequence, structure or expression) similarity score for each pair of lncRNAs in the group and the average score of the group. We also calculated a similarity score for each pair of protein isoforms that have interaction with the lncRNAs in the group and the average score of all such pairs of protein isoforms. The similarity score between two lncRNA (or protein isoform) sequences is defined as the global alignment score normalized by the alignment length. All similarity scores were normalized to the range of [0, 1]. At last, Pearson’s correlation coefficient (PCC) was used to measure the pairwise correlation between lncRNA sequence similarity and protein isoform sequence similarity (Fig. 3a). Similarly, we calculated the PCC between lncRNA expression similarity and protein isoform expression similarity (Fig. 3b), and the PCC between lncRNA structure similarity and protein isoform structure similarity (Fig. 3c). The PCC between lncRNAs sequence similarity and lncRNA expression similarity (Fig. 3d) and the PCC between protein isoform sequence similarity and isoform expression similarity (Fig. 3e) are also included as a useful reference.

Clearly, positive correlations are found in all above analyzes. The strong correlations in Fig. 3a–c conform that similar lncRNAs tend to interact with similar protein isoforms. An interesting observation is that our correlation analysis results are highly consistent with the experimental results in subsection . For example, the strongest correlation between the sequence similarities of lncRNAs and protein isoforms is consistent with the most significant drop in the prediction performance when the sequence data was removed. The moderate correlation coefficients in Fig. 3d, e suggest that the sequence and expression data contain complementary features and thus might explain why their combination helped improve the performance of our model.

#### Performance on an independent interaction dataset of mouse.

To further validate the effectiveness of DeepLPI in lncRNA-protein interaction prediction, we test DeepLPI and the other existing methods on a dataset independent from the training data. More specifically, we trained all models with the human lncRNA-protein interactions from the NPinter v3.0 database and tested the models on 3580 mouse lncRNA-protein interactions in the same database. Although there is a high genetic similarity between mouse and human (and hence the conservation of lncRNAs), the performance of all models dropped. The AUC of DeepLPI decreased from 0.866 (human) to 0.753 (mouse), but it was still the best since the highest AUC of the other models on the mouse test data was 0.68. An obvious reason for the performance drops might be because lncRNAs do not show the same pattern of evolutionary conservation as protein-coding genes [50].

To further investigate the prediction performance of DeepLPI on interactions between proteins and lncRNAs conserved between human and mouse such as Gas5, Rmst, Neat1 and Meg3 [50, 51], we selected 39 interactions involving conserved lncRNAs from the 3580 mouse interactions. Of the 39 interactions, 89.7% have been correctly predicted by DeepLPI. In particular, since Gas5 is an extensively studied mouse lncRNA that plays an important role in modulating self-renewal [52], we show the interaction prediction results concerning mouse Gas5 in Additional file 1: Table S1. The table demonstrates again that DeepLPI achieved the highest prediction accuracy.

#### A case study on new interactions

We further validate our model using some new lncRNA-protein interactions from the recent literature that were not included in the NPinter v3.0 database. After a careful literature search, we found 12 new lncRNA-protein interactions [53–56]. The details of these interactions are provided in Additional file 1: Table S2. The prediction results concerning these new lncRNA-protein interactions by the methods are illustrated in Additional file 1: Table S3. The results show that DeepLPI was able to find out novel interactions often missed by the other methods.

## Conclusion

The knowledge of interactions between lncRNAs and protein isoforms could help understand the functions of lncRNAs. In this paper, we proposed a machine-learning based method, DeepLPI, to predict interactions between lncRNAs and protein isoforms. DeepLPI uses a multimodal deep learning neural network to extract intrinsic features from the sequence and structure data of lncRNAs and protein isoforms and a conditional random field to extract network topological features from their expression data. We designed a multiple instance learning iterative algorithm to train the prediction model using an availablelncRNA-protein interaction dataset, and performed extensive experiments to show that DeepLPI achieves a significantly better accuracy in predicting lncRNA-protein interactions compared with the state-of-the-art methods. The multimodal learning feature of DeepLPI allows it to integrate more types of data besides sequences, structures and expression profiles. With minor modifications, DeepLPI could be adapted to predict miRNA-protein interactions, as well as more complex interactions such as lncRNA-miRNA-protein interactions.

Our divergence analysis shows that many isoforms of the same gene interact with different lncRNAs. Hence, it would be of practical importance to study the interactions between lncRNAs and protein isoforms (as opposed to proteins or genes). However, as far as we know, DeepLPI is the first attempt to predict lncRNA-protein isoform interactions, and its performance is still far from being desirable. It might be possible to improve the performance of DeepLPI by using better (e.g., tissue-specific) expression data, more detailed protein secondary structure representations and high quality isoform-isoform interaction network data. We plan to investigate these directions in the near future.

## Methods

### Datasets

The ground truth interactions between lncRNAs and proteins were downloaded from the NPInter v3.0 database [42]. This is the most enriched database that integrates experimentally verified functional interactions. We kept only the interacting pairs labeled with ‘Homo sapiens’. Though the data of NPInter has kept on growing, the number of involved lncRNAs and proteins is still very small at present. In the current version, there are 10031 interactions between 1817 lncRNAs and 151 proteins. These interactions are considered as positive interactions. To train a neural network model, we also need to sample negative interactions that represent pairs of lncRNAs and proteins that do not interact with each other. As the population of negative interactions count is large, complete random sampling of it may contain few lncRNAs and proteins that present in positive interactions, which might lead to overfitting [57]. To reduce overfitting, we make sure that at least a half of the negative lncRNA-protein interaction pairs contain lncRNAs and proteins that appear in positive interaction pairs (but do not interact with each other). The rest of the negative interaction pairs consist of randomly chosen lncRNAs and proteins that do not appear in positive interaction pairs.

The lncRNA sequences and the protein isoform sequences of human genome were downloaded from GENCODE [58] and ENSEMBL [59], respectively. The sequences were then used to predict their secondary structures. To predict the secondary structure of an lncRNA, we used RNAShapes [60]. The output of RNAShapes was converted to a structure sequence using the EDeN tool (http://github.com/fabriziocosta/EDeN) as in [61]. An lncRNA structure typically consists of six structural components: stem (S), multiloop (M), hairpin loop (H), internal loop (I), dangling end (T), and dangling start (F). To predict the secondary structure from a protein isoform sequence, we used SPIDER2 [62]. SPIDER2 uses a deep neural network to predict a 3-state protein secondary structure whose structural components consist of helix (H), strand (E) and coil (C).

The third type of data that we collected are mRNA and lncRNA expression data. The mRNA expression data are obtained from the literature [49], and the lncRNA expression data were downloaded from the Co-LncRNA database [63]. The mRNA and lncRNA expression data are based on high-throughput human RNA sequencing experiments of 334 studies (1,735 samples) and 241 studies (6,560 samples), respectively. We used the expression data to build co-expression networks. To ensure network quality, we only considered RNA sequencing studies with at least ten samples. Finally, 42 mRNA sequencing studies and 54 lncRNA sequencing studies were kept with a total of 1134 samples and 1429 samples, respectively. Note that an mRNA transcript uniquely corresponds to a protein isoform. In the following, an isoform means either an mRNA transcript or protein isoform. Since different databases use different identifier naming conventions to record protein isoforms, mRNA and lncRNA, ID conversion tools from [63–65] were used to identify the same moleculars from different data sources and perform the mapping between protein isoforms and mRNAs. Finally, we filtered the data and kept the isoforms and lncRNAs that appear in both the sequence data and the expression data.

#### Data representation

An lncRNA is a character sequence composed of 4 unique ribose nucleotides: cytosine (C), adenine (A), guanine (G), and uracil (U). A protein isoform is a sequence consisting of 20 unique amino acid codes. We generate hexamers and trimers from an lncRNA sequence and a protein isoform sequence, respectively. An lncRNA of length *n* is represented as $n-5$ consecutive hexamers of ribose nucleotides, and a protein isoform of length *n* is represented as $n-2$ consecutive trimers of amino acids. A hexamer of nucleotides is encoded as an integer from 0 to $4^6-1$, and a trimer of amino acids is encoded as an integer from 0 to $20^3-1$. As in [66], to help our deep learning model to learn the intrinsic properties of the sequences efficiently, the integer encoding sequences of lncRNAs and proteins are further encoded using a standard dense embedding technique [67]. A dense embedding maps an integer index of the vocabulary to a dense vector of floats, which is achieved by an embedding layer of our deep learning network using the training data. The embedding layer aims to obtain meaningful dense vectors, which could be utilized to calculate correlations between sequences and are used as the input features of lncRNA and protein isoforms. We used a 64-dimensional dense vector to encode a hexamer of nucleotides (or a trimer of amino acids).

**Fig. 4:**
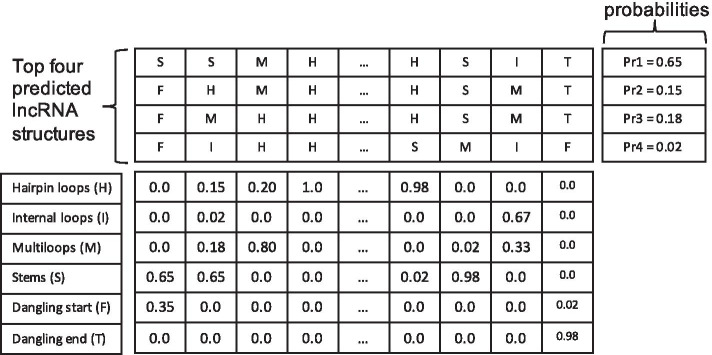
The representation of multiple predicted structures of an lncRNA. Four predicted structures are merged into a single matrix based on their probabilities

**Fig. 5:**
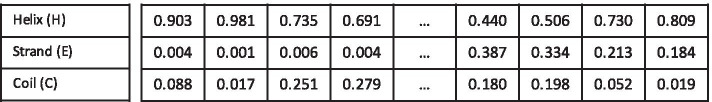
The representation of multiple predicted structures of a protein. SPIDER2 predicts the probability of each candidate structure, which is summed into a matrix according to structural component types and amino acid positions

Different from the sequence data, the structure of an lncRNA or a protein is often not unique, since multiple structures could be predicted for a single sequence by RNAShapes and SPIDER2. To keep more predicted structural information of an lncRNA of length *n*, a $6 \times n$ matrix as shown in Fig. 4 is used to encode multiple predicted structures, where the six rows represent six different structural component types and the value at the *i*th row and *j*th column is the sum of probabilities of the predicted structures with the *j*th nucleotide of the lncRNA being of the *i*th structural component type. Similar to the lncRNA structure representation, a $3 \times n$ probability matrix as shown in Fig. 5 is used to represent multiple predicted structures of SPIDER2 for a protein with *n* amino acids.

### Model architecture and training

**Fig. 6:**
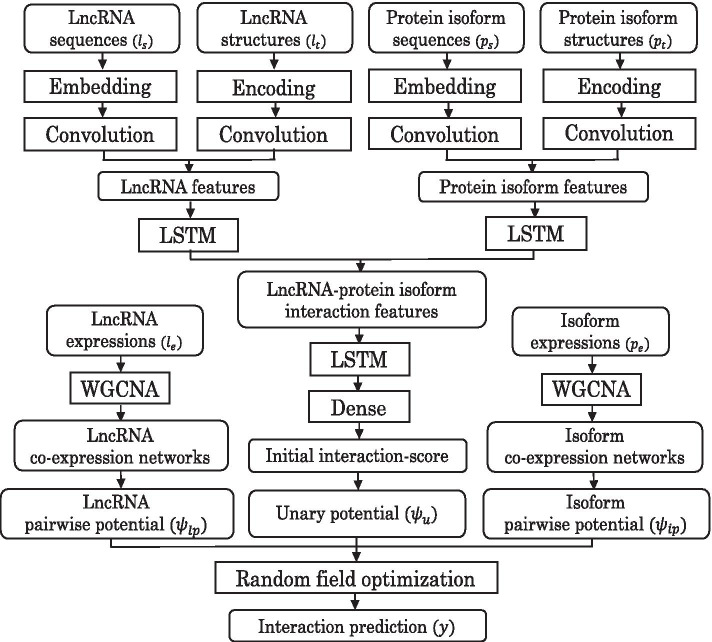
A flowchart of DeepLPI. It begins with a multimodal deep learning neural network (MDLNN) that uses embedding layers, convolutional layers, LSTM layers and other layers of Keras to extract features from the sequence and structure data of lncRNAs and protein isoforms, and calculate initial interaction scores. Weighted correlation network analysis (WGCNA) is used to construct co-expression networks from expression data of lncRNAs and protein isoforms. Based on the pairwise potentials and unary potentials inferred from the co-expression relationship and the initial interaction scores, respectively, a conditional random field (CRF) optimization is used to predict the interactions between lncRNAs and protein isoforms. The whole model is trained using an iterative semi-supervised learning algorithm based on multiple instance learning (MIL)

DeepLPI predicts the interactions between lncRNAs and protein isoforms by integrating the information of sequence, structure and expression data into a unified predictive model. It consists of two learning submodels. The first is a multimodal deep learning neural network (MDLNN) model and the second a conditional random field (CRF) model. The MDLNN model extracts and fuses the (intrinsic) features from the sequence and structure data of lncRNAs and protein isoforms, and calculates the initial scores of the interactions between lncRNAs and protein isoforms. The CRF model makes a final prediction based on both the initial interaction scores and the expression data of mRNAs (corresponding to protein isoforms) and lncRNAs. To overcome the lack of ground truth interactions between lncRNAs and proteins, we develop a semi-supervised algorithm following [39–41] to train the MDLNN and CRF models together iteratively. Figure 6 shows a schematic illustration of DeepLPI. More details of the method are described in the following subsections.

#### Extracting sequence and structure features using multimodal deep learning neural network

To learn intrinsic features related to lncRNA-protein isoform interactions from the sequence and structure data, we construct a multimodal deep learning neural network (MDLNN). We use convolutional layers to extract local features and long short-term memory layer (LSTM) layers to extract short-range and long-range dependencies. At first, MDLNN uses a standard dense embedding technique [67] to map the sequences of lncRNAs and protein isoforms into a 64-dimensional vector space, which is implemented by using embedding layers (denoted as embed(.)) of Keras [68]. After a training process, the embedding layers are able to learn appropriate mappings such that the mapped dense vectors could capture similarities between the sequences. Then, the dense vector matrices representing the sequences and the matrices encoding the predicted structures of lncRNAs and protein isoforms pass through one-dimensional convolutional layers with 4 convolutional filters (denoted as conv(.)) to obtain the local features of the sequence and structure data. After that, max pooling (denoted as pool(.)) layers are used to downsample the output of the convolutional layers to reduce the learning time of the subsequent layers. Based on downsampled features, LSTM layers (denoted as lstm(.)) are used to learn the features that represent the short-range and long-range intrinsic properties of the sequences and structures as in [69, 70]. These features extracted from lncRNA sequences, lncRNA structures, protein isoform sequences and structures are merged together as the input of an LSTM layer followed by a dense layer. The LSTM layer and dense layer (denoted as dense(.)) are intended to learn the interaction patterns between lncRNAs and protein isoforms. Finally, the output of the dense layer is fed into a logistic regression layer (denoted as logit(.)) to compute an initial interaction score. Given an lncRNA sequence $l_s$, a protein isoform sequence $p_s$, and the predicted structures $l_t$ and $p_t$ of the lncRNA sequence and protein isoform sequence, respectively, the initial interaction score (IIS) is calculated as follows:

1 $$\begin{aligned}&{\text{IIS}}(l_s, p_s, l_t, p_t) \\&={\text{logit(dense(lstm(merge}}(f_{l}(f_{l_s}(l_s), f_{l_t}(l_t)), f_{p}(f_{p_s}(p_s), f_{p_t}(p_t)))))) \\&f_{p}(f_{p_s}(p_s), f_{p_t}(p_t)) = {\text{lstm(merge}}(f_{p_s}(p_s), f_{p_t}(p_t))) \\&f_{l}(f_{l_s}(l_s), f_{l_t}(l_t)) = {\text{lstm(merge}}(f_{l_s}(l_s), f_{l_t}(l_t))) \\&f_{l_s}(l_s) = {\text{pool(conv(embed}}(l_s))) \\&f_{p_s}(p_s) = {\text{pool(conv}}((p_s))) \\&f_{l_t}(l_t) = {\text{pool(conv(embed}}(l_t))) \\&f_{p_t}(p_t) = {\text{pool(conv}}((p_t))) \\ \end{aligned}$$

#### Incorporating co-expression relationships using a CRF

Based on the experimental evidence that we have found in the literature, co-expressed isoforms and co-expressed lncRNAs often exhibit similar interactions [71]. To incorporate the co-expression relationships between the isoforms and between the lncRNAs, we use a weighted correlation network analysis (WGCNA) method [72] to construct a co-expression network for the isoforms and one for the lncRNAs separately. In the lncRNA (or protein isoform) co-expression network, the vertices are the lncRNAs (or isoforms, respectively). The edge between vertices *i* and *j* has weight $w_{i j} = s_{i j}^\beta$, where $s_{i j}$ is the absolute value of the Pearson correlation coefficient (PCC) between the expression profiles of the corresponding lncRNAs (or isoforms) and $\beta$ is the soft thresholding parameter ($\beta = 6$ in our experiments as suggested by [73] for unsiged networks). Based on the pairwise potentials inferred from the co-expression relationships and the unary potentials inferred from the initial interaction scores output by the MDLNN, DeepLPI next uses a conditional random field (CRF) optimization to predict the interactions between lncRNAs and protein isoforms. Note that our CRF optiimzation framework is very similar to the framework introduced in [39] for inferring isoform functions. Since many details are different, we will still include a full description of it below for completeness.

For the *i*th lncRNA-protein isoform pair, denote the lncRNA sequence as $l_{s_i}$, the protein isoform sequence as $p_{s_i}$, the lncRNA structure as $l_{t_i}$, the protein isoform structure as $p_{t_i}$, the lncRNA expression profile as $l_{e_i}$, the protein isoform expression profile as $p_{e_i}$, and the binary label indicating whether there is an interaction between the lncRNA and the protein isoform as $y_i$. The CRF optimization model aims to obtain the labels *y* for each lncRNA-protein isoform pair by minimizing the Gibbs energy function below:

2 $$\begin{aligned} E(y|l_s, p_s, l_t, p_t, l_e, p_e) = \theta _1\sum _{i}\psi _u(y_{i}|l_{s_{i}}, p_{s_{i}}, l_{t_{i}}, p_{t_{i}}) + \\ \theta _2\sum _{i<j}\psi _{ip}(y_{i}, y_{j} | p_{e_{i}}, p_{e_{j}}) + \theta _3\sum _{i<j}\psi _{lp}(y_{i}, y_{j} | l_{e_{i}}, l_{e_{j}}) \\ \end{aligned}$$

Here, the Gibbs energy is a weighted summation of unary potentials $\psi _u$, isoform pairwise potentials $\psi _{ip}$ and lncRNA pairwise potentials $\psi _{lp}$. The unary potentials $\psi _u$ are calculated from the the initial interaction scores as $\psi _u(0|l_{s_{i}}, p_{s_{i}}, l_{t_{i}}, p_{t_{i}}) = {\text{IIS}}(l_s, p_s, l_t, p_t)$ and $\psi _u(1|l_{s_{i}}, p_{s_{i}}, l_{t_{i}}, p_{t_{i}}) = 1 - {\text{IIS}}(l_s, p_s, l_t, p_t)$. For the *i*th and *j*th lncRNA-protein isoform pairs, their pairwise potential is defined as follows:

3 $$\begin{aligned} \psi _{ip}(y_{i}, y_{j} | p_{e_{i}}, p_{e_{j}}) = \mu _{p}(y_{i}, y_{j})\sum _{q}w_q(p_{e_{i}}, p_{e_{j}}) \\ \psi _{lp}(y_{i}, y_{j} | l_{e_{i}}, l_{e_{j}}) = \mu _{p}(y_{i}, y_{j})\sum _{r}w_r(l_{e_{i}}, l_{e_{j}}) \end{aligned}$$

where $w_q(p_{e_{i}}, p_{e_{j}})$ is the weight of the edge between isoforms *i* and *j* in the *q*-th isoform co-expression network and $w_r(p_{e_{i}}, p_{e_{j}})$ is the weight of the edge between lncRNAs *i* and *j* in the *r*-th lncRNA co-expression network. $\mu _{p}(y_{i}, y_{j})$ is a label compatibility function whose value is 1 if $y_i \ne y_j$ or 0 otherwise. It is used to penalize highly co-expressed isoforms and highly co-expressed lncRNAs assigned with different interaction labels. The weights $\theta _1$, $\theta _2$ and $\theta _3$ are used to control the relative importance of $\psi _u$, $\psi _{ip}$ and $\psi _{lp}$ in the Gibbs energy. They will be discussed in the next subsection.

By searching for an assignment ${\hat{y}}$ of labels minimizing the Gibbs energy $E({\hat{y}}|l_s, p_s, l_t, p_t, l_e, p_e)$, we attempt to find appropriate labels for lncRNA-protein isoform pairs with low unary energies such that highly co-expressed isoforms would have the same interaction patterns with highly co-expressed lncRNAs. Since computing an exact solution to the Gibbs energy minimization problem is challenging, we apply an efficient approximation algorithm called the mean-field approximation as in [74] to obtain an approximate solution, sketched below.

It is easy to see that minimizing the Gibbs energy is equal to maximizing the following probability:

4 $$\begin{aligned} P(y|l_s, p_s, l_t, p_t, l_e, p_e) = \frac{1}{Z}exp(-E(y|l_s, p_s, l_t, p_t, l_e, p_e)) \end{aligned}$$

where $Z= \sum _y exp(-E(y|l_s, p_s, l_t, p_t, l_e, p_e))$ is a normalization constant. Let $Q(y|l_s, p_s, l_t, p_t, l_e, p_e)$ be the product of independent marginal probabilities, i.e.,

5 $$\begin{aligned} Q(y|l_s, p_s, l_t, p_t, l_e, p_e) = \prod _i Q_i(y_{i}|l_{s_{i}}, p_{s_{i}}, l_{t_{i}}, p_{t_{i}}, l_{e_{i}}, p_{e_{i}}) \end{aligned}$$

Instead of computing the exact distribution of $P(y|l_s, p_s, l_t, p_t, l_e, p_e)$, we use $Q(y|l_s, p_s, l_t, p_t, l_e, p_e)$ with the minimum KL-divergence **D**(*Q*||*P*) to approximate *P*, and adopt the following iterative update equation to obtain a *Q* with the minimum KL-divergence:

6 $$\begin{aligned}&Q_i(y_{i}|l_{s_{i}}, p_{s_{i}}, l_{t_{i}}, p_{t_{i}}, l_{e_{i}}, p_{e_{i}}) = \frac{1}{Z_i}~exp \{-\theta _1 \psi _u(y_{i}|l_{s_{i}}, p_{s_{i}}, l_{t_{i}}, p_{t_{i}}) \\&\quad - \theta _2 \sum _{i\ne j} \sum _q w_q (p_{e_{i}}, p_{e_{j}}) Q_j(1-y_i|l_{s_{j}},p_{s_{j}},l_{t_{j}},p_{t_{j}},l_{e_{j}},p_{e_{j}}) \\&\quad - \theta _3 \sum _{i\ne j} \sum _r w_r (l_{e_{i}}, l_{e_{j}}) Q_j(1-y_i|l_{s_{j}},p_{s_{j}},l_{t_{j}},p_{t_{j}},l_{e_{j}},p_{e_{j}})\} \end{aligned}$$

Here, we initialize $Q_i$ with the unary potential and update it iteratively according to Eq. 6 until convergence, when the final output of our model is obtained.

#### Training the model with the MIL framework

Because the ground truth lncRNA-protein isoform interactions are generally unavailable, conventional supervised training algorithms cannot be directly applied to our model. Similar to [39] again, here we adopt a semi-supervised training algorithm under the MIL framework as in [40, 41]. In this MIL framework, for each lncRNA, a protein/gene is treated as a bag, the isoforms of a protein/gene are treated as the instances in the bag, and only the ground truth of the bag (i.e., the true lncRNA-protein interaction label) is assumed. We further require that a positive bag should contain at least one positive instance and a negative bag should contain no positive instances. DeepLPI first initializes all instances of positive bags with positive labels, and the other instances with negative labels. Then, the model parameters are optimized with the initial labels in the following standard supervised learning manner.

Given a batch of training instances $(l_{s},p_{s},l_{t},p_{t},l_{e},p_{e}, {\hat{y}})$, the loss functions in terms of the MDLNN parameters *w* and in terms of the CRF parameters $\theta$ are defined as the following negative log likelihoods, respectively.

7 $$\begin{aligned} \ell _{{{\text{MDLNN}}}} \,(w:l_{s} ,p_{s} ,l_{t} ,p_{t} ,\hat{y}) & = - \sum\limits_{i} {(\hat{y}_{i} \log ({\text{IIS}}(l_{{s_{i} }} ,p_{{s_{i} }} ,l_{{t_{i} }} ,p_{{t_{i} }} ))} \\ & \quad + (1 - \hat{y}_{i} )\log (1 - {\text{IIS}}(l_{{s_{i} }} ,p_{{s_{i} }} ,l_{{t_{i} }} ,p_{{t_{i} }} ))) \\ \end{aligned}$$

8 $$\ell _{{{\text{CRF}}}} \,(\theta :l_{s} ,p_{s} ,l_{t} ,p_{t} ,l_{e} ,p_{e} ,\hat{y}) = - \log P(\hat{y}|l_{s} ,p_{s} ,l_{t} ,p_{t} ,l_{e} ,p_{e} ) + \sum\limits_{i} {\frac{{\theta _{i}^{2} }}{{2\sigma ^{2} }}}$$

In Eq. 8, the parameter $\sigma$ is used to regularize the importance of the co-expression networks in the model optimization and set as 0.1 in our following experiments. We use the Nadam optimization algorithm to update the MDLNN parameters *w* so $\ell _{\mathrm{MDLNN}}$ could be minimized. To minimize $\ell _{\mathrm{CRF}}$, we use the L-BFGS-B algorithm as in [39] to update CRF parameters $\theta$.

We perform inference for every instance in the positive bags after each update of the parameters of the model, using the model with the updated parameters. Here, the label of an instance is updated according to the inference: ${\hat{y}}_i={\text{argmax}}_{y_i} P_i(y_i)$. We also adopt the following constraint: for each positive bag, if all its instances are assigned with negative labels, we force the instance with the largest positive prediction score $P_i(1)$ in the bag as positive. The steps of updating parameters and labels are repeated alternately until convergence.

## Supplementary Information

**Additional file 1**. Supplementary Materials

## Data Availability

DeepLPI is implemented in Python and freely available to the public on https://github.com/dls03/DeepLPI

## Abbreviations

LncRNAs: Long non-coding RNAs
PPI: Protein–protein interaction
DNN: Deep neural network
MDL: Multimodal deep learning
CRF: Conditional random field
MIL: Multiple instance learning
AUC: Area under receiver operating characteristic curve
AUPRC: Area under precision-recall curve
MDLNN: Multimodal deep learning neural network
LSTM: Long short-term memory layer
IIS: Initial interaction score
WGCNA: Weighted correlation network analysis
PCC: Pearson correlation coefficient
LOOCV: Leave-one-out cross-validation
MIPs: Proteins with multiple isoforms
