# Supplementary material for: DeepLPI: a multimodal deep learning method for predicting the interactions between lncRNAs and protein isoforms
Source: BMC Bioinformatics. 2021 Jan 18;22:24. doi: 10.1186/s12859-020-03914-7 (PMC7814738; doi:10.1186/s12859-020-03914-7)
Supplement: Supplementary file 1 — Additional file 1. Supplementary Materials [file 12859_2020_3914_MOESM1_ESM.pdf]

DeepLPI: a multimodal deep learning method for predicting the interactions between lncRNAs and protein isoforms (Supplementary Materials)

Dipan Shaw[1], Hao Chen[1], Xie Minzhu[2], and and Tao Jiang[1,3]

[1] Department of Computer Science and Engineering, University of California, Riverside, CA 92521, USA
[2] College of Information Science and Engineering, Hunan Normal University, Changsha, China
[3] Bioinformatics Division, BNRIST / Department of Computer Science and Technology, Tsinghua University, Beijing, China

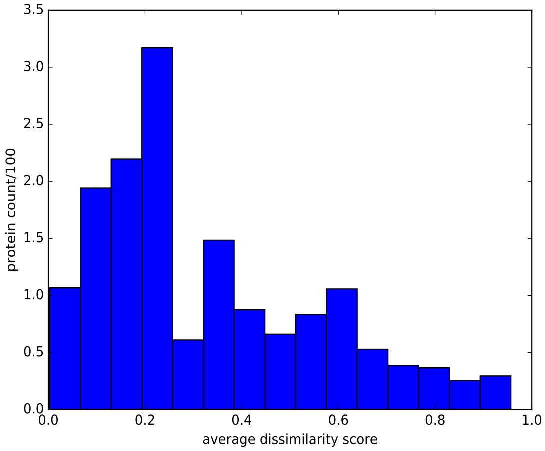

Fig. S1: Distributions of semantic dissimilarity scores of MIPs. For each MIP, the semantic dissimilarity score indicates the divergence of the lncRNAs interacting with its different isoforms. The score range of [0, 1] is equally divided into 15 bins. For each bin, we count how many MIPs have semantic dissimilarity scores in this range.

Table S1: Prediction of interactions involving mouse lncRNA Gas5.

lncRNA	Protein	Machine-learning based methods							Network based methods				
		lncPro	IPMiner	lncADeep	rpiCool	RPITER	LPI-BLS	DeepLPI	LPIHN	LPBNI	LPLNP	PLIPCOM	SFPEL-LPI
Gas5	Q92900	o	o	o	x	o	x	o	o	o	o	o	o
Gas5	Q92833	o	o	o	x	o	x	o	o	o	o	o	o
Gas5	Q8BIF2	x	x	o	x	o	x	o	x	x	x	x	o
Gas5	Q16630	x	x	x	x	x	x	o	x	x	x	x	o
Gas5	Q9NR56	x	x	x	x	x	x	x	x	x	x	x	o
Gas5	Q8R003	o	x	o	x	o	o	o	x	x	x	o	x
Gas5	P38432	x	x	o	x	o	o	o	x	o	o	o	x
Gas5	P84103	x	o	o	x	o	o	o	x	o	o	o	x
Gas5	Q08170	x	x	o	x	o	o	o	o	x	x	o	x
Gas5	Q15910	x	o	o	x	o	o	o	x	x	x	x	x
Gas5	H0YB86	x	o	x	o	x	o	o	o	o	o	x	o
Gas5	P35637	x	x	x	o	o	x	o	x	o	x	o	o
Gas5	Q921F2	x	x	o	o	x	x	o	x	x	o	x	o
Recall		.231	.385	.692	.231	.615	.462	.923	.385	.462	.462	.538	.615

Note: The predicted and unpredicted interactions are represented as circles and crosses in the table.

Table S2: The source of 12 recently reported lncRNA-protein interactions in the literature.

**lncRNA**	**Protein**	**Reference**
PANDAR	PTBP1	Found in paper Pospiech *et al.* (2018)
lnc-SH2D7.1	FBXL22	Found in paper Zhang *et al.* (2018)
lnc-SH2D7.1	LPIN2	Found in paper Zhang *et al.* (2018)
lnc-DCAF811.1	PEBP1	Found in paper Zhang *et al.* (2018)
lnc-DCAF811.1	DNAJB12	Found in paper Zhang *et al.* (2018)
lnc-NIT1	POR	Found in paper Zhang *et al.* (2018)
AC011498.1	CEBPA	Found in paper Yin *et al.* (2018)
CRNED	CEBPA	Found in paper Yin *et al.* (2018)
LINC00504	CEBPA	Found in paper Yin *et al.* (2018)
AC011498.1	NPM1	Found in paper Yin *et al.* (2018)
LL22NC03-N64E9.1	KLF2	Found in paper Xing *et al.* (2018)
LL22NC03-N64E9.1	EZH2	Found in paper Xing *et al.* (2018)

Table S3: Prediction results concerning 12 new lncRNA-protein interactions from recent literature.

lncRNA	Protein	Machine-learning based methods							Network based methods				
		lncPro	IPMiner	lncADeep	rpiCool	RPITER	LPI-BLS	DeepLPI	LPIHN	LPBNI	LPLNP	PLIPCOM	SFPEL-LPI
PANDAR	PTBP1	x	o	o	o	o	o	o	x	x	x	x	x
lnc-SH2D7.1	FBXL22	x	o	x	x	o	x	o	o	x	x	x	x
lnc-SH2D7.1	LPIN2	o	x	o	o	x	o	o	o	o	o	o	o
lnc-DCAF811.1	PEBP1	o	x	x	x	o	o	o	o	o	o	o	o
lnc-DCAF811.1	DNAJB12	o	x	x	x	o	x	o	x	o	o	x	o
lnc-NIT1	POR	o	x	o	x	x	x	o	x	o	x	x	o
AC011498.1	CEBPA	x	x	x	x	o	x	x	x	x	x	x	x
CRNED	CEBPA	x	o	o	x	x	o	x	x	x	x	x	x
LINC00504	CEBPA	x	o	o	o	o	o	o	x	x	x	x	o
AC011498.1	NPM1	x	x	o	o	x	x	o	x	x	x	o	x
LL22NC03-N64E9.1	KLF2	x	x	x	x	o	x	x	x	x	o	o	x
LL22NC03-N64E9.1	EZH2	x	o	o	x	o	o	o	x	o	x	o	o
Recall		.333	.417	.583	.333	.666	.500	.750	.250	.417	.333	.417	.500

Note: The predicted and unpredicted interactions are represented as circles and crosses in the table.

Bibliography

Pospiech, N., Cibis, H., Dietrich, L., Müller, F., Bange, T., and Hennig, S. (2018). Identification of novel pandar protein interaction partners involved in splicing regulation. *Scientific reports*, **8**(1), 2798.

Xing, Y., Zhao, Z., Zhu, Y., Zhao, L., Zhu, A., and Piao, D. (2018). Comprehensive analysis of differential expression profiles of mrnas and lncrnas and identification of a 14-lncrna prognostic signature for patients with colon adenocarcinoma. *Oncology reports*, **39**(5), 2365–2375.

Yin, X., Huang, S., Zhu, R., Fan, F., Sun, C., and Hu, Y. (2018). Identification of long non-coding rna competing interactions and biological pathways associated with prognosis in pediatric and adolescent cytogenetically normal acute myeloid leukemia. *Cancer cell international*, **18**(1), 122.

Zhang, M., Gu, Y., Su, M., Zhang, S., Chen, C., Lv, W., and Zhang, Y. (2018). Inferring novel lncrna associated with ventricular septal defect by dna methylation interaction network. *BioRxiv*, page 459677.
